# Burden and severity of inherited monoamine neurotransmitter rare genetic disorders in India

**DOI:** 10.1186/s13023-026-04309-2

**Published:** 2026-03-27

**Authors:** Runa Hamid, Vykuntaraju K. Gowda, Lloyd Tauro, Rakesh Mishra

**Affiliations:** 1 Tata Institute for Genetics and Society, Bengaluru, Karnataka India; 2 Department of Pediatric Neurology, Indira Gandhi Institute of Child Health, Bengaluru, Karnataka India

**Keywords:** Monoamine neurotransmitter disorders, Tyrosine hydroxylase, Aromatic L-amino acid decarboxylase, GTPCH1, Tetrahydrobiopterin, Oculogyric crisis, Dopamine, Serotonin, Rare genetic disorders, Hyperphenylalaninemia

## Abstract

Monoamine neurotransmitter metabolic disorders (mNMDs) are a diverse group of rare genetic conditions caused by disruptions in the metabolism of catecholamines (dopamine, epinephrine, and norepinephrine) and serotonin. These disorders predominantly affect children, often manifesting as neurodevelopmental and mental health challenges. Despite their clinical significance, there is a conspicuous lack of comprehensive reviews focused on the Indian population. The prevalence and distribution of mNMDs within India remain largely unexplored, underlining an urgent need for systematic data collection and analysis. This review aims to address this knowledge gap by compiling individual case reports of mNMDs documented in India, identifying genetic factors contributing to these conditions, and describing their manifestations. It provides an overview of diagnosed cases of mNMDs from various hospitals across the country, emphasizing the current limitations of diagnostic tools and the need for specialized testing. By illuminating mNMDs occurrence and clinical characteristics in India, this review seeks to encourage greater awareness about these disorders in India and the need to develop effective diagnostic and therapeutic strategies.

## Introduction

Rare Genetic Disorders (RGDs) represent a diverse group of conditions resulting from genetic mutations that affect a very small fraction of the population. Despite their rarity, RGDs collectively have significant implications for public health. Current data reveal that approximately 18% of protein-coding genes are implicated in RGDs, with the Central Nervous System (CNS) being predominantly affected. Notably, 74% of single-gene RGDs documented in the Online Mendelian Inheritance in Man (OMIM) database are associated with CNS involvement [1]. These findings underscore the profound impact of RGDs, particularly those affecting the CNS. This burden affects healthcare systems globally, including countries with resource-limited settings like India.

**Fig. 1:**
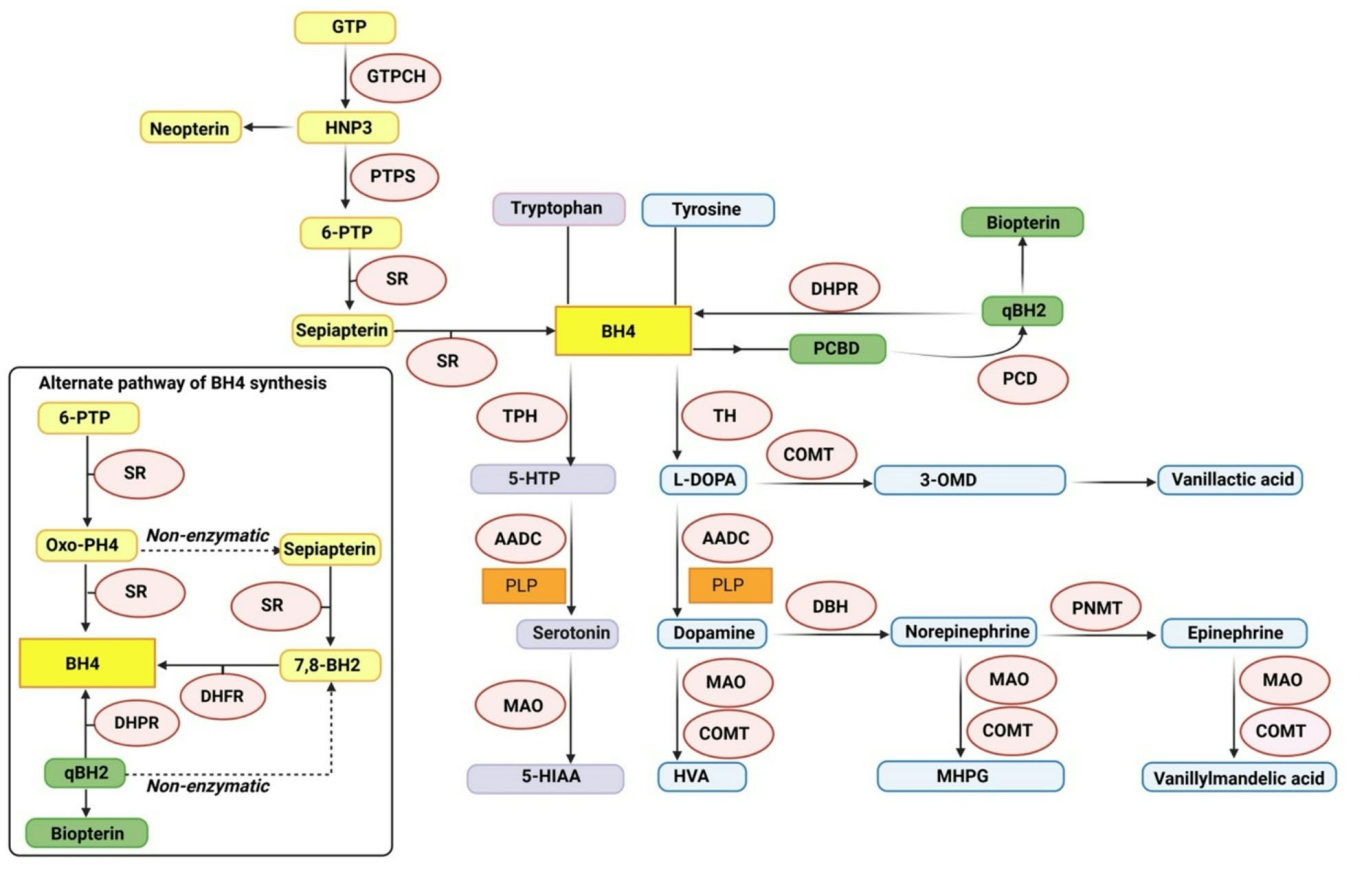
Monoamine neurotransmitter metabolic pathway. Overview of monoamine neurotransmitter synthesis and metabolism in neurons. Tyrosine and tryptophan are converted into monoamines—dopamine, norepinephrine, epinephrine, and serotonin through sequential enzymatic reactions involving key enzymes: GTPCH 1, PTPS, SR, DHPR, PCD, TpH, TH, AADC, DBH and PNMT (Red ovals). Essential cofactors include BH4 (yellow box) and PLP (orange box). Metabolism of these neurotransmitters occurs via MAO and COMT, yielding major end-products: HVA, MHPG, Vanillylmandelic acid (VMA) (blue boxes) and 5-HIAA (purple box). BH4 is synthesized via the de novo pathway (through 6-PTP and Oxo-PH4) or alternatively via the salvage pathway (via sepiapterin and 7,8-BH2), and the recycling pathway (via quinonoid form of BH2, qBH2).The table categorizes the key metabolites, enzymes and Cofactors from the pathway

Among the RGDs, mNMDs are a distinctive group of inherited metabolic disorders affecting the synthesis, cofactor availability, transport and degradation of catecholamine neurotransmitters (dopamine, epinephrine, and norepinephrine) and serotonin- the key neurotransmitters of the CNS. Based on the kind of molecular defect, mNMDs are categorized into four groups: (1) defects in monoamine biosynthesis (2) defects in cofactors (3) defects in monoamine transporters (4) defects in monoamine catabolism (Fig. 1).

The first category includes defects due to monoamine biosynthesis enzymes: Tyrosine hydroxylase (TH) deficiency, Tryptophan hydroxylase (TpH) deficiency and Aromatic L-aminoacid decarboxylase (AADC) deficiency. These disorders impair dopamine and serotonin synthesis, are inherited in an autosomal recessive manner and characterized by spectrum of motor phenotypes such as uncoordinated walking (ataxia), dystonia, oculogyric crisis, hypotonia, developmental delay with varying degree of severity from person to person. TH deficiency is caused by *TH* gene mutations and also referred to as Segawa syndrome, dopa-responsive dystonia, infantile parkinsonism or tyrosine hydroxylase deficient dopa-responsive dystonia. TpH deficiency is caused due to mutations in *TpH1* and *TpH2* genes The disorder is characterized by impaired serotonin synthesis affecting all the physiological and CNS related functions facilitated by serotonin. AADC deficiency is caused due to mutations in dopa decarboxylase gene (*DDC* gene) leading to combined dopamine and serotonin deficiency. This deficiency is also referred as DDC deficiency.

The second category of mNMD disorders involves defects in essential cofactors: Tetrahydrobiopterin (BH4) and Pyridoxal 5’ phosphate (PLP). BH4 deficiencies are further subdivided according to the specific enzyme defects involved in its biosynthesis: GTP cyclohydrolase 1 (GTPCH1); pyruvoytetrahydropterin synthase (PTPS); sepiapterin reductase (SR) and recycling enzymes: Pterin-4a carbinolamine dehydratase (PCD) and Dihydropterin reductase (DHPR). Clinical manifestations include dystonia, tremors, ataxia, oculogyric crisis, uncontrolled movements of head and neck, cognitive deficits, diurnal variations and occasionally microcephaly. All deficiencies are inherited in autosomal recessive manner except for GTPCH deficiency which can be inherited as autosomal dominant or autosomal recessive. The recessive form of GTPCH deficiency is associated with more severe symptoms as compared to the dominant form of the disorder. SR deficiency leads to only cerebral BH4 deficiency because BH4 produced elsewhere in the body does not require SR. During the BH4 recycling, PCD converts BH4 into BH2 which is further recycled into BH4 by DHPR activity. During PCD deficiency, the synthesis of BH4 is affected and the condition is associated with Hyperphenylalanemia (HPA). PLP deficiency is another cofactor defect caused due to mutations in *PNPO* gene which disrupts vitamin B6 metabolism and AADC function, often associated with neonatal seizures and encephalopathy.

The third category involves disorders due to defects in monoamine transporters- Dopamine transporter (DAT) and Vesicular monoamine transporter (VMAT2). DAT and VMAT2 regulates neurotransmitter reuptake and vesicular storage, respectively. DAT is caused by mutations in *SLC6A3* gene resulting in infantile-onset parkinsonism-dystonia with tremors, rigidity and progressive motor function. VMAT2 deficiency is caused due to mutations in *SLC18A2* gene, also leading to parkinsonism like features commonly called as infantile-onset parkinsonism- dystonia 2, epilepsy and oculogyric crisis. Both DAT and VMAT2 are recessively inherited.

The fourth category includes disorders due to defects in monoamine catabolism involving Dopamine-ß-hydroxylase gene *(DBH gene)* encoding DBH enzyme, an autosomal recessive disorder and impairs conversion of dopamine into norepinephrine. The symptoms include sharp drop in blood pressure, droopy eyelids and inability to stand for long hours, muscle weakness and behavioral changes. Monoamine oxidase A (MAO-A) deficiency impairs the MAO-A enzyme that catalyzes the oxidation of monoamine neurotransmitters. It is an X linked raregenetic disorder, also known as Brunner syndrome. The disorder is common in males and characterized by anti-social, aggressive and impulsive behaviors often overlapping with autism spectrum disorder and ADHD. PNMT is another enzyme in the pathway whose mutation impairs conversion of norepinephrine to epinephrine and is the final step in the epinephrine synthesis.

The diagnosis of mNMDs relies on combination of clinical phenotypic evaluation, biochemical profiling of CSF and other body fluids, therapeutic response and genetic analysis. Among these, clinical presentation and response to levodopa is an important diagnostic indicator. Patients with GTPCH1 deficiency have been reported to show dramatic response to levodopa [2, 3]. Also, TH, SR and PTPS deficiencies have also been reported to show varying degrees of responsiveness to levodopa [4–11]. In situations where genetic or biochemical testing is not available, levodopa administration is a strong indicator of underlying monoamine neurotransmitter defect. Cerebrospinal fluid (CSF) biogenic amine metabolite analysis of HVA, HIAA and 3-OMD is also indicative of diagnosis [12, 13]. CSF pterin analysis can also provide diagnostically interpretable patterns [14]. HPA or Phenylketonuria (PKU) is caused due to abnormally high levels of phenylalanine and is another diagnostic clue for mNMDs. HPA may also arise due to mutations in genes involved in the BH4 generation and regeneration [15]. PKU test is a routine test performed in newborn screening. Worldwide data of newborn screening shows prevalence of PKU with an average of 1:10,000 and out of these, BH4 deficiencies constitute around 1–2% of monoamine disorder cases [16]. Therefore, enhanced levels of phenylalanine is considered as one of the primary diagnostic hallmarks of BH4 deficiencies. Urine pterin profiling offers a non-invasive diagnostic approach for mNMDs. Enzyme defects in BH4 pathway result in accumulation of pterins and their derivatives- Neopterin, Biopterin, Dihydrobiopterin, Sepiapterin. Profiling of these metabolites in DBS, Serum and urine samples have been used to differentiate BH4 deficiencies from HPA [17]. CSF biochemical analysis has also been used for mNMD diagnosis and is considered as a gold standard test globally with genetic testing as supportive confirmatory test. However, CSF analysis requires a lumbar puncture, an invasive procedure often avoided in children with nonspecific symptoms, resulting in further diagnostic delays.

This review provides a retrospective data and analysis of reported mNMD cases in India, emphasizing the increasing prevalence of these disorders across country. As these conditions are potentially treatable and manageable conditions, the work underscores the importance of newborn screening for early detection and treatment, which can significantly improve patient outcomes. By consolidating available reported cases, we aim to highlight the growing identification of the patients, the diagnostic challenges faced by them, and therapeutic interventions currently followed in the country.

## Clinical reports of mNMDs from India

The following case-reports describe the clinical details of mNMDs patients, and their genetic findings are summarized in the accompanying table (Table 1). The characteristic features, diagnostic approaches employed, and some of the therapeutic interventions being practiced in each subtype has also been summarised (Table 2):

**Table 1:** Case-reports of monoamine neurotransmitter deficiencies in Indian patients

Deficiency and Gene name	Patient	cDNA position	Amino acid change	Exon number	Reference
**Tyrosine Hydroxylase deficiency (** ***TH*** ** gene) **	P1	c.1196 C > T (Pathogenic) c.- 2188_103-194del c.16G > A; p. Ala6Thr (Non-Path.)	p. Thr399Met - p. Ala6Thr	11 1 1	[18]
	P2	c.698G > G/A	p.R233H	6	[6]
	P3	c.1282G > G/A	p.G428R	12	[6]
	P4	c.1282G > G/A	p.G428R	12	[6]
	P5	Genetic Mutation N.D *^1&2^			[19]
	P6	Genetic Mutation N.D *^1&2^			[19]
	P7	Genetic Mutation N.D *^1&2^			[19]
	P8	Genetic Mutation N.D *^1&2^			[20]
	P9-P12	Genetic Mutation N.D *^1,2&3^			[21]
	P13	Genetic Mutation N.D *^1&2^			[22]
	P14	Genetic Mutation N.D *^1&2^			[23]
	P15	Genetic Mutation N.D *^1&2^			[24]
	P16	Genetic Mutation N.D *^1&2^			[25]
	P17	c.698G>A	p.Arg233His		[26]
	P18	c.457C>T c.919T > G	p.Arg153Ter p.Ser307Ala	4 8	Information courtesy Dr Vykuntaraju Gowda
	P19	c.1103C>T	p.Thr368Met	10	Information courtesy Dr Vykuntaraju Gowda
	P20	c.977C>T c.1228C>T	p.Pro326Leu p.Arg410Trp	8 12	Information courtesy Dr Vykuntaraju Gowda
	P21	c.793C >T	p.Arg265Trp	7	Information courtesy Dr Vykuntaraju Gowda
	P22	c.1009C>T	p.Pro337Cys	9	[27]
	P23	c.1207T>C	p.Phe403Leu	11	[27]
**Total**	**23**				
**Aromatic L-Aminoacid decarboxylase deficiency ** **(** ***DDC*** **gene)**	P24	c.1060G>T	p.Gly354Cys	12	[8]
	P25	c.1072C>T c.140C>A (Compound Heterozygous)	p.Arg358Cys p.Pro47His	12 2	[8]
	P26	c.1072C>T c.140C>A (Compound Heterozygous)	p.Arg358Cys p.Pro47His	12 2	[8]
	P27	c.140C>A	p.Pro47His	2	[8]
	P28	c.140C>A	p.Pro47His	2	[8]
	P29	c.208C>T	p.His70Tyr	3	[8]
	P30	c.175G>A	p.Asp59Asn	2	[8]
	P31	c.475G>A	p.Ala159Thr	5	[28]
	P32	c.475G>A	p.Ala159Thr	5	[28]
	P33	Genetic Mutation N.D *^1&3^			[29]
	P34	c.1040G>A	p.Arg347Gln	11	Information courtesy Dr Vykuntaraju Gowda
	P35	Genetic Mutation N.D *^1^			Information courtesy Dr Vykuntaraju Gowda
	P36	c.175G>A	p.Asp59Asn	2	[27]
**Total**	**13**				
**GTP Cyclohydrolase 1 deficiency (** ***GCH 1*** ** gene)**	P37	c.703C>G	p.Arg235Gly	3	[7] Reported on BioPKU also
	P 38	c.457C>T	p.His153Tyr	3	[10]
	P 39	c.703C>G	p.Arg235Gly	6	[10]
	P40	Genetic Mutation N.D*^1&2^			[30]
	P41	Genetic Mutation N.D*^1&2^			[30]
	P42	c.734T>C	p.Leu245Pro	6	[27]
**Total**	**6**				
**Pyruvoyltetrahydropterin synthase deficiency (** ***PTS*** ** gene)**	P43	c.65 C>G	p.Ala22Gly	1	[10]
	P44	c.G>T (genomic coordinate unclear in the report)	p.D116Y	6	[31]
**Total**	**2**				
**Sepiapterin reductase deficiency (** ***SPR*** ** gene) **	**P4** ***5***	c.413T>A	p.V138D	Not mentioned	[11]
	**P4** *6*	c.413T>A	p.V138D	Not mentioned	[11]
	**P4** **7**	c.177delT	p.Gly60Ala	1	[11]
	**P48 (**Sibling of P4**7**)	Genetic Mutation N.D*^1,2&3^			[11]
	**P49**	c.544C>T	p.Gln182Ter	2	Information courtesy Dr Vykuntaraju Gowda
	**P50**	c.655C>T	p.Arg219Ter	3	Information courtesy Dr Vykuntaraju Gowda
**Total**	**6**				
**Dihydropteridine reductase deficiency (** ***QDPR*** ** gene)**	**P51**	c.488G>T	p.Ser163lle	Not mentioned	[32]
	**P52**	c.635T>C	p.Phe212Ser	7	[11]
	**P53**	c.488G>T	p.Ser163Ile	5	[10]
	**P54**	c.68G>A	p.Gly23Asp	1	[10]
	**P55**	c.295 + 5G>T	5’ splice site	Intron 3	[10]
	**P56**	c.680T> C	p.Leu227Pro	7	[10]
	**P57**	c.296-1G>T	3’ splice site	Intron 3	[10]
	**P58**	c.635T> C	p.Phe212Ser	7	[10]
	**P59**	c.661C>T	p.Arg221*	7	[32]
**Total**	**9**				
**Pyridoxal 5’ phosphate deficiency (** ***PNPO*** ** gene)**	**P60**	c.482G>A	p.Arg161His	5	[33]
	**P61**	c.352G>A	p. Gly118Arg	3	[34]
	**P62**	c.413G>A	p. Arg138His		[35]
	**P63**	c.412C>T	p.Arg138Cys	4	Information courtesy Dr Vykuntaraju Gowda
	**P64**	c.224T>G	p.Ile75Arg	2	Information courtesy Dr Vykuntaraju Gowda
	**P65**	c.413G>A	p.Arg138His	4	Information courtesy Dr Vykuntaraju Gowda
**Total**	**6**				
**Dopamine transporter deficiency (** ***SLC6A3 *** **gene)**	**P66**	c.1105G>A	p.Gly369Arg	8	[27]
**Total**	**1**				
**Vesicular monoamine transporter deficiency (** ***SLC18A2 *** **gene)**	**P67**	c.1196 A>C	p.Asp399Ala	14	[36]
(Three Indian patients out of 42 total patients included in the study Source: as per personal communication with authors)	**P68**	c.710C>G	p.Pro237Arg	7	[37]
	**P69**	c.1253A>G	p.Tyr418Cys	14	[37]
	**P70**	C992-3C>T	3’ Splice site	Intron-10 (VUS)	[37]
**Total**	**4**				

N.D Not determined

*^1^Diagnosis done based on symptoms

^2^Diagnosis done based on response to Levodopa

^3^ Diagnosis done based on biochemical tests

**Table 2:** mNMDs with causative gene, clinical features, and treatment strategies

Primary monoamine neurometabolic disorder	Gene	Symptoms	Age of symptom onset	Therapeutic interventions
AADC deficiency	*DDC*	Hypotonia, abnormal rotation of eyes (oculogyric crises), diurnal variations	2 months	**Drug therapy**: Vitamin B6, dopamine agonists, and MAO inhibitors **Gene therapy**: FDA recently approved first gene therapy for AADC to PTC Therapeutics, Inc.
GTPCH1 deficiency	*GCH1*	Severe developmental retardation, severe muscular hypotonia of the trunk and hypertonia of the extremities, convulsions, and autonomic disturbance	5 months	Levodopa-carbidopa supplementation
PTPS deficiency	*PTS*	Hypotonia and delayed motor development	4 months	Tetrahydrobiopterin (BH4), L-DOPA, and 5-HTP supplementation
DHPR deficiency	*QDPR*	Hypotonia, dystonia, tremors, diurnal variations, and seizures		Sapropterin therapy/ Tetrahydrobiopterin (BH4) supplementation
SR deficiency	*SPR*	Diurnally fluctuating movement disorder is usually associated with cognitive delay and severe neurologic dysfunction	7 months	Levodopa and 5-HTP supplementation
PLP deficiency	*PNPO*	Neonatal-onset severe seizures and subsequent encephalopathy		Pyridoxal phosphate supplementation
TH deficiency	*TH*	Uncoordinated walking (gait ataxia), involuntary muscle contraction (dystonia)	5 months	Low-dose L-DOPA and selegiline
DβH deficiency	*DBH*	Severe orthostatic hypotension, ptosis, nasal stuffiness, impaired ejaculation, and a neonatal history of delayed eye opening		DOPA supplementation
VMAT deficiency	*SLC18A2*	Abnormal movements, including parkinsonism, dystonia, and poor fine motor skills, as well as autonomic dysfunction, including abnormal sweating, cold extremities, and poor sleep		Dopamine agonist
DAT deficiency	*SLC6A3*	Hyperkinesia with orolingual and limb dyskinesia, dystonia, and chorea, or hypokinesia with Parkinsonian features, such as bradykinesia, rigidity, and tremor	5–7 months	NA
MAOA deficiency	*MAOA*	Impulsive aggressiveness and mildly impaired intellectual development	Childhood	Selective serotonin reuptake inhibitor (SSRI) drug therapy
COMT deficiency	*COMT*	Schizophrenia, hallucinations, delusions, social and occupational deterioration, disorganized speech, and catatonic behavior	Late adolescence	NA

### TH deficiency

23 pediatric patients with age range from 6 month to 15 years (P1 to P23), in addition to a 24 year old patient (P17) with prolonged episodic dystonia with onset at the age of 2 years [26] have been reported to be affected by TH deficiency [6, 18–26, 38]. Almost all patients presented with motor symptoms including gait abnormalities with diurnal variation. Additional features included abnormal limb posture with truncal deviation to one side of the body, and progressive walking difficulties associated with limb stiffness were also reported in these patients. Some individuals showed delayed attainment of developmental milestones since birth, while others initially experienced difficulties with fine motor tasks such as writing, which gradually increased over time. In majority of the cases the final diagnosis was based on clinical features and their improvement with levodopa therapy. WES and CSF analysis was also performed for few of the cases. Management of TH deficiency primarily involved long-term levodopa therapy which led to clinical improvement in most patients.

### TpH deficiency

To the best of our knowledge, currently, there are no specific published case reports from India. Globally, the documented cases are rare.

### AADC deficiency

We identified case reports of 13 patients (P24 to P36) with age range from 5 months to 10 years to be affected by AADC deficiency [8, 28, 29, 38]. Predominant features were development delay, hypotonia, oculogyric crises, dystonia and diurnal variation of symptoms. Diagnosis was based on characteristic clinical feature with supportive biochemical CSF analysis in some cases or genetic analyses. Treatment and management of symptoms in majority cases involved therapy with pyridoxal 5’-phosphate, anticholinergic and dopamine agonists with variable response to drugs ranging from partial to good improvements.

### GTPCH1 deficiency

6 Indian patients (P37 to P42) with age range from 8 months to 5 years have been reported to be affected by GTPCH1 deficiency [7, 10, 30]. The patients presented with developmental delay, oculogyric crisis, intermittent dystonia, hypotonia. A couple of patients also showed tremors and motor defect. The diagnosis was mainly through genetic analysis and tandem mass spectrometry by quantifying phenylalanine levels. Management of symptoms were through therapy with levodopa, carbidopa, phenylalanine restricted diet and tryptophan supplementation which resulted in functional gains, with many achieving improved ambulation.

### PTPS deficiency

2 patients- 9 months and 7 years old (P43-P44) have been reported with developmental delay and typical motor symptoms like tremors or dystonia [10, 31]. Diagnosis was suggested based on response to levodopa and confirmed through genetic analysis. Biochemical abnormalities of BH4 pathway were also reported. The therapy and management of the symptoms were through treatment with phenylalanine restricted diet, levodopa with Carbidopa or tryptophan supplementation.

### SR deficiency

6 patients (P45 to P50) from age range 11 months to 10 years were reported for SR deficiency in India [11, 29, 38]. Major features reported were psychomotor retardation, floppy state, oculogyric crisis, dystonia, uncontrolled movements of head and neck, cognitive deficits, sleep disturbances and diurnal variations. Diagnosis was through CSF biochemical analysis or genetic analysis. Symptoms were managed through L-DOPA/Carbidopa supplements. One of the patients showed excellent developmental progress attaining age-appropriate milestones following levodopa/carbidopa with tryptophan.

### DHPR deficiency

9 patients (P51 to P59) with age range from 3 months to 2 years were reported for DHPR deficiency in India [10, 29, 32, 38]. They presented typical clinical symptoms including developmental delay, seizures, oculogyric crisis, dystonia, hypotonia. One patient also showed ataxia while one other presented microcephaly. Diagnosis was through elevated phenylalanine, elevated ratio of phenylalanine to tyrosine, and genetic analysis. Almost all patients were treated and managed with doses of levodopa, folinic acid and phenylalanine restricted diet and marked improvement were reported.

### PLP deficiency

6 patients (P60 to P65) with age range from 6 months to 12 years have been reported for PLP deficiency in India [33–35]. They presented clinical symptoms of seizures, encephalopathy, severe developmental delay and dystonia. In all the cases diagnosis was through genetic analysis. The patients were treated and managed by administering pyridoxal phosphate supplements and combination therapy with anti-seizure drugs [27].

### DAT deficiency

Only one patient (P66) of 4 years was reported with DAT deficiency [38]. Symptoms involved developmental delay, seizures, microcephaly, strabismus, spasticity, clonus, and brisk reflexes. Diagnosis was through genetic analysis. Clinical suspicion arose from the siblings’ presentation with abnormal posturing and hypertonia. Management was done through levodopa along with other supportive medication. The improvement was limited, and patient was reported to demonstrate mild symptomatic improvement.

### VMAT2 deficiency

4 Indian patients (P67 to P70) from age 4 months to 1 year were reported with VMAT2 deficiency due to *SLC18A2* gene mutation [36, 37]. Also referred to as infantile-onset parkinsonism-dystonia-2. Early infancy symptoms were reported with hypokinesia, tremors, dystonia, oculogyric crises, epilepsy and occasionally microcephaly. In all the patient’s, diagnosis was through genetic analysis. A broader study describing *SLC18A2 *variants affecting 42 individuals in 27 families from across different countries in the world including from India were reported. The number of patients from India which are included in the study are not mentioned in the article. However, as per the personal communication with one of the authors, there were three Indian patients (P68 to P70) included in the study [37]. Therapeutic benefits were not much observed in the patients.

### DßH deficiency

No confirmed case reports from India were identified. However, a number of population-based studies performed in Indian population highlights the *D*ß*H* variants in context of different neurological and psychiatric disorders like Schizophrenia, Parkinson’s, ADHD etc [39–41].

### MAO and COMT deficiency

Few studies in different Indian cohorts have examined association of MAO variants with ADHD [42, 43] and COMT variant with Schizophrenia in a cohort of North Indian population [44]. Prevalence of both MAO and COMT in India is unknown. We could not find any case-report describing the pathogenic variants in Indian patients for both these genes.

### PNMT deficiency

To the best of our knowledge, none of the cases of PNMT deficiency have been reported in India.

Collectively, all these clinical reports illustrate that almost all categories of mNMDs have been identified and reported in India. India is the most populated country of the world and the true burden of these disorders is unknown which is expected to be much higher than reported.

## Diagnostic challenges associated with mNMDs in the Indian context

As reported in the case-studies from India, clinically, mNMDs have a broad spectrum of phenotypes including hypotonia, dystonia, developmental delay, oculogyric crisis, sleep disorders, microcephaly, dyskinesia, epileptic seizures etc. Diagnosis is often underrecognized or misdiagnosed in the country because the symptoms overlap with other neurological problems like cerebral palsy, encephalopathies etc. Awareness regarding mNMDs is limited among the general population as well as in the practicing physicians. This results in delayed diagnosis, misdiagnosis and consequent under-reporting. There are no universally standard diagnostic tests for these disorders leading to inconsistent use of certain diagnostic parameters which increases the likelihood of misdiagnosis and potential for missing the accurate underlying condition. In general, there is a frequent diagnostic reliance on levodopa responsiveness. Absence of diagnostic facilities for CSF biogeneic amine analysis is unavailable at most centres in the country posing a major limitation. Moreover, financial and resource constraints of several families to genetic testing leads to compounding of the problem. Collectively, all these limitations underscores the urgent need for the development of a simple non-invasive, standardized and cost-effective diagnostic test which can reliably identify the majority of disorders within this group and enable timely targeted therapeutic interventions.

## Discussion

mNMDs represent a clinically important and treatable subgroup of metabolic rare genetic disorders affecting monoamine neurotransmitters. Globally, a comprehensive data on mNMDs has been compiled by International working group on Neurotransmitter-Related Disorders (iNTD) which reports hundreds of case across multiple countries worldwide. In 2025, PD Hinduja Hospital, Mumbai, India, became part of this consortium reflecting a growing recognition of mNMDs in the country. An international survey of patients with HPA associated BH4 deficiencies identified 626 patients, of which 2.1% patients were Indians suggesting India contributes to a quantifiable fraction of global cases [15]. India-specific information on RGDs related to neurotransmitters is also available in the recently launched GenTIGS database [45]. From the compiled Indian reports of 70 patients in this work, almost all mNMDs have been identified in the Indian population. Biosynthesis pathway and cofactor defects are the most frequently reported whereas catabolic pathway and transporter defects majorly remain undocumented which is likely due to poor diagnostic accessibility in the country rather than absence of cases. Given India’s largest population, high birth rate, absence of a widespread newborn screening program and consanguineous marriages being common in several communities, the burden is expected to be much higher than reported.

Clinically, the major occurrence of mNMDs in India is in the paediatric group between 0 and 10 years of age with several cases also reported in young adolescents (between 10 and 16 years of age). Common clinical features reported among Indian patients include hypotonia, dystonia, oculogyric crisis, diurnal variation along with occasionally reported seizures, microcephaly and sleep disturbances. The major phenotypes observed are in concordance with the ones previously reported globally [46]. Importantly, as per reports, oculogyric crisis, dystonia, diurnal variations and levodopa responsiveness in the Indian patients are the critical indicators of mNMDs. A consistent theme reported across almost all Indian reports is misdiagnosis or delayed diagnosis. Misdiagnosis is generally reported due to clinical overlap with cerebral palsy, encephalopathy and developmental disorders. CSF analysis which is considered the gold standard globally is available only in couple of centres in India. Genetic testing, although increasingly accessible, is financially prohibitive to majority families. Since previous surveys demonstrate that BH4 disorders constitute about approximately 1–2% of PKU (HPA) patients globally [15], a systematic newborn screening program in India can facilitate identification of BH4 disorder subgroup of treatable mNMDs.

One of the most important characteristic observed in mNMDs is the responsiveness to therapeutic interventions, atleast in several subtypes of this group. However, it is notable that therapeutic success is dependent on timely intervention because delayed diagnosis may lead to irreversible deficits. Therefore, mNMDs represent an unique group of underrecognized rare genetic disorders in the country, which are treatable and early interventions can dramatically alter quality of life. Hence these warrants attention in order to reduce the impact on the healthcare system of the country.

## Conclusion and the way forward in Indian context

Several previous studies report disorder-specific metabolic patterns characterized by accumulation of upstream substrates and reduction of downstream metabolites corresponding to the enzymatic block or non-functional cofactors and have explored peripheral fluids (dried blood spots, plasma or urine) and CSF to analyze monoamine metabolites with a diagnostic perspective [15, 47–59]. Although CSF is a preferred mode for diagnosis, plasma can present a more patient-friendly alternative and would enhance testing, early screening, and allow for longitudinal therapeutic monitoring without subjecting the patient to risks associated with CSF collection. To consolidate available evidence, a table has been included that summarizes metabolite alterations in mNMDs highlighting both reported accumulations/reductions and the expected biochemical patterns (Table 3). However, future comparative studies are required to evaluate whether peripheral fluids reliably reflects biochemical changes in CSF. The application of multiplexing and sensitive technologies like LC-MS/MS can be one of the promising methods to quantify multiple metabolites in a single assay to achieve differential diagnosis across mNMDs. Considering the risk-benefit balance associated with invasive CSF collection and its unavailability in Indian centers versus peripheral fluid sampling on dried blood spots, development of multiplexed quantitative assay in peripheral fluids could be a cost-effective and high-impact diagnostic strategy in the Indian context.

**Table 3:** Expected metabolic patterns in different monoamine neurotransmitter rare genetic disorders and compiled information on metabolites reported as increased or decreased in blood in different studies

Primary monoamine neurometabolic disorder	Expected modulated monoamine metabolites		Modulated monoamine metabolites reported in blood		Matrix	References
	Accumulation	Deficiency	Accumulation	Deficiency		
AADC deficiency	↑L-DOPA ↑3-OMD ↑5-HTP	↓HVA ↓VMA ↓MHPG ↓5- HIAA	↑L-DOPA ↑3-OMD ↑5-HTP	↓HVA ↓VMA ↓MHPG ↓5- HIAA	Blood	[53] [56] [57]
			↑3-OMD	**-**	DBS	[47]
GTPCH1 deficiency	↑Phenylalanine	↓Neopterin ↓Biopterin ↓Sepiapterin ↓BH4 ↓5- HIAA ↓HVA ↓VMA ↓MHPG ↓L-DOPA ↓3-OMD ↓5-HTP	↑Phenylalanine	↓Neopterin ↓Biopterin ↓Serotonin	Blood	[58] [59]
			↑Phenylalanine	-	DBS	[59]
PTPS deficiency	↑Phenylalanine ↑Neopterin	↓Sepiapterin ↓BH4 ↓L-DOPA ↓3-OMD ↓5-HTP ↓5-HIAA ↓HVA ↓MHPG ↓VMA	↑Phenylalanine	-	Blood	[15] [52]
			↑Phenylalanine ↑Neopterin	-	DBS	[51]
DHPR deficiency	↑Phenylalanine ↑BH2 ↑Biopterin	↓5- HIAA ↓HVA ↓VMA ↓MHPG ↓L-DOPA ↓3-OMD ↓5-HTP	↑Phenylalanine ↑Neopterin	↓Biopterin	Blood	[51] [58]
			↑Phenylalanine	-	DBS	[15]
SR deficiency	↑Sepiapterin ↑Biopterin ↑BH2	**-**	**-**	**-**	**-**	**-**
PLP deficiency	↑L-DOPA ↑5-HTP	↓5-HIAA ↓HVA ↓MHPG ↓VMA	**-**	**-**	**-**	**-**
TH deficiency	↑Tyrosine	↓HVA ↓VMA ↓MHPG ↓L-DOPA ↓3-OMD	**-**	**-**	**-**	**-**
DβH deficiency	↑Dopamine ↑HVA	↓MHPG ↓VMA	**-**	**-**	**-**	**-**
VMAT deficiency	↑HVA ↑5- HIAA ↑3-OMD ↑VMA ↑MHPG ↑VLA	**-**	**-**	**-**	**-**	**-**
DAT deficiency	↑HVA ↑5- HIAA ↑3-OMD ↑VMA ↑MHPG ↑VLA		**-**	**-**	**-**	**-**
MAO A deficiency	↑Serotonin	↓5-HIAA	**-**	-	**-**	**-**
COMT deficiency	↑Dopamine ↑Norepinephrine ↑Epinephrine	↓HVA ↓MHPG ↓VMA	**-**	**-**	**-**	**-**

As discussed in the preceding sections, mNMDs are rare genetic disorders that are potentially treatable and manageable upon timely interventions. Despite the growing recognition of mNMDs, their diagnosis in India remains a significant challenge due to limited awareness among clinicians, absence of uniform diagnostic assessment method, availability of CSF analysis centres, lack of mandatory newborn screening programs, financial constraints of patients for genetic testing. These limitations lead to underdiagnosis and potential misdiagnosis, delaying appropriate treatment for affected individuals. Given the importance of early and accurate diagnosis in improving patient outcomes, there is a pressing need to develop standardized uniform diagnostic assays in the country for this group of disorders. Disorder specific metabolite pattern assessment in body fluids may serve as a valuable diagnostic indicator. In addition, a second level test for BH4 deficiencies should be integrated in newborn screening programs for relevant biomarkers and be made mandatory for HPA positive patient. All these could be crucial steps toward improving the identification and management of these disorders in India.

## Data Availability

Not applicable.
